# Identification, Cloning, and Characterization of l-Phenylserine Dehydrogenase from *Pseudomonas syringae* NK-15

**DOI:** 10.4061/2010/597010

**Published:** 2010-03-25

**Authors:** Sakuko Ueshima, Hisashi Muramatsu, Takanori Nakajima, Hiroaki Yamamoto, Shin-ichiro Kato, Haruo Misono, Shinji Nagata

**Affiliations:** ^1^The United Graduate School of Agricultural Science, Ehime University, 3-5-7 Tarumi, Matsuyama 790-8566, Japan; ^2^Faculty of Agriculture, Kochi University, B200 Monobe, Nankoku, Kochi 783-8502, Japan; ^3^Corporate Research Center, R&D Management, Daicel Chemical Industries, Ltd., 1239 Shinzaike, Aboshi-ku, Himeji 671-1283, Japan; ^4^Himeji Research Center, Daicel Chemical Industries, Ltd., 1239 Shinzaike, Aboshi-ku, Himeji 671-1283, Japan; ^5^Science Research Center, Kochi University, B200 Monobe, Nankoku, Kochi 783-8502, Japan

## Abstract

The gene encoding d-phenylserine dehydrogenase from *Pseudomonas syringae* NK-15 was identified, and a 9,246-bp nucleotide sequence containing the gene was sequenced. Six ORFs were confirmed in the sequenced region, four of which were predicted to form an operon. A homology search of each ORF predicted that *orf3* encoded l-phenylserine dehydrogenase. Hence, *orf3* was cloned and overexpressed in *Escherichia coli* cells and recombinant ORF3 was purified to homogeneity and characterized. The purified ORF3 enzyme showed l-phenylserine dehydrogenase activity. The enzymological properties and primary structure of l-phenylserine dehydrogenase (ORF3) were quite different from those of d-phenylserine dehydrogenase previously reported. l-Phenylserine dehydrogenase catalyzed the NAD^+^-dependent oxidation of the *β*-hydroxyl group of l-*β*-phenylserine. l-Phenylserine and l-*threo*-(2-thienyl)serine were good substrates for l-phenylserine dehydrogenase. The genes encoding l-phenylserine dehydrogenase and d-phenylserine dehydrogenase, which is induced by phenylserine, are located in a single operon. The reaction products of both enzymatic reactions were 2-aminoacetophenone and CO_2_.

## 1. Introduction

**Scheme 1:**
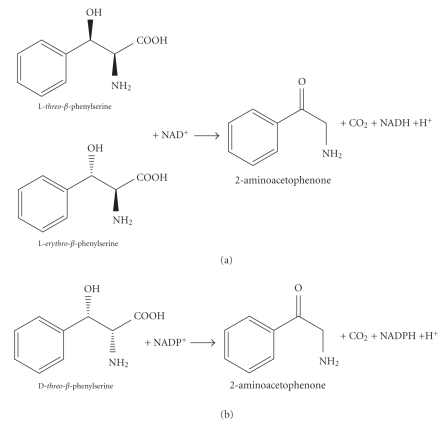
Reactions catalyzed by l-phenylserine dehydrogenase (a) and d-phenylserine dehydrogenase (b).

3-Hydroxy-2-amino acids are components of many bioactive molecules, such as antibiotics and immunosuppressants [1–9] and a drug for Parkinson's disease therapy [10]. Therefore, enzymatic synthesis of 3-hydroxy-2-amino acids with d- and l-threonine aldolases has been performed extensively [1, 2, 4–9]. *β*-Phenylserine (2-amino-3-hydroxy-3-phenylpropanoic acid), which exists as four stereoisomers, is one of the physiologically important 3-hydroxy-2-amino acids [11–13]. However, until recently, little was known about phenylserine biosynthetic and degradation pathways. To elucidate metabolic processes involving phenylserine, we have attempted to obtain enzymes physiologically acting on phenylserine. Previously, we reported the molecular characteristics of inducible pyridoxal 5'-phosphate (PLP)-dependent phenylserine aldolase [EC  4.1.2.26] [14], PLP-dependent phenylserine dehydratase [EC  4.2.1.-] [15], and inducible NADP^+^-dependent d-phenylserine dehydrogenase [EC  1.1.1.-] (Scheme 1) [16]. During the identification of the gene encoding d-phenylserine dehydrogenase, we found the gene encoding l-phenylserine dehydrogenase [EC  1.1.1.-] in the same operon. In this paper, we report the identification and cloning of the genes encoding d-phenylserine dehydrogenase and l-phenylserine dehydrogenase. Moreover, the enzymological properties of l-phenylserine dehydrogenase (Scheme 1) overexpressed in *Escherichia coli* are described. 

## 2. Materials and Methods

### 2.1. Materials

d-*threo*-*β*-Phenylserine was a gift from Mr. Teruyuki Nikaido, Daicel Chemical Industries (Hyogo, Japan). Polypepton was from Nihon Pharmaceutical (Tokyo, Japan). NAD^+^, NADP^+^, yeast extract, and molecular-weight marker-proteins for gel filtration were from Oriental Yeast (Tokyo, Japan). Restriction enzymes and kits for genetic manipulation were from Takara Shuzo (Kyoto, Japan), Toyobo (Osaka, Japan), and New England Biolabs (Beverly, MA). All other reagents were of analytical grade from Sigma (St. Louis, MO), Nacalai Tesque (Kyoto, Japan), and Wako Pure Chemical Industries (Osaka, Japan).

### 2.2. Cultivation

*Pseudomonas syringae* NK-15 was cultivated at 30°C in a medium containing 0.5% dl-*threo*-*β*-phenylserine, 1.5% polypepton, 0.2% K_2_HPO_4_, 0.2% KH_2_PO_4_, 0.2% NaCl, 0.01% MgSO_4_ *·*7H_2_O, and 0.01% yeast extract (pH 7.2) with reciprocal shaking [16].

### 2.3. Determination of Internal Amino Acid Sequence

Purified d-phenylserine dehydrogenase, prepared as previously described [16], was lyophilized and suspended in 8 M urea. After incubation for 1 hour at 37°C, the enzyme was digested with lysyl endopeptidase for 15 hours at 37°C. The resultant peptides were separated on a Shimadzu HPLC system equipped with a YMC-Pack C4 column (6 × 150 mm, YMC Co., Kyoto, Japan) using a solvent system of 0.1% trifluoroacetic acid (solvent A) and acetonitrile containing 0.07% trifluoroacetic acid (solvent B). A 90-min linear gradient from 5 to 50% solvent B was used to elute peptides at a flow rate of 1.0 ml/min. The absorbance at 210 nm of the effluent was continuously monitored. The internal amino acid sequence of d-phenylserine dehydrogenase was determined using an automated protein sequencer (Perkin Elmer, Wellesley, MA).

### 2.4. Identification of the Gene Encoding d-Phenylserine Dehydrogenase and Gene Organization

Based on the N-terminal amino acid sequence of d-phenylserine dehydrogenase, determined as described previously [16], and the internal amino acid sequence of the enzyme determined in this work, inverse PCR was performed to identify the gene encoding d-phenylserine dehydrogenase. PCR products were sequenced with an Applied Biosystems 373A DNA sequencer and a DNA sequencing kit (ABI PRISM Dye Terminator Cycle Sequencing Ready Reaction Kit). Inverse PCR was also used to determine the nucleotide sequence of the regions upstream and downstream of the d-phenylserine dehydrogenase gene.

### 2.5. Cloning and Expression of the Gene Encoding d-Phenylserine Dehydrogenase and the Orf3 Gene in *Escherichia coli*

Chromosomal DNA was prepared from *P. syringae* NK-15 by the method of Saito and Miura [17]. A DNA fragment containing the gene encoding d-phenylserine dehydrogenase was amplified by PCR with Ex *Taq* DNA polymerase (Takara Shuzo, Kyoto, Japan) using a sense primer containing an *Eco*RI site (5′- GCGGAATTCGAATCCGCCAACCCACGCCAAGGAATAACGCA -3′) and an antisense primer containing a *Pst*I site (5′- GCGAAGCTTCTGCAGCAAGCAGCGCTCACGTCGAAGCGCACA-  3′). The amplified DNA fragment was ligated into the *Eco*RI-*Pst*I site of pUC18. The resultant plasmid, pUPsDH, was introduced into *E. coli* JM109 to provide recombinant d-phenylserine dehydrogenase. *E. coli* JM109 carrying pUPsDH was cultivated in LB medium containing 50 *μ*g/ml ampicillin and 0.1 mM isopropyl-*β*-d-thiogalactopyranoside (IPTG) at 37°C for 20 hours. A DNA fragment containing the *orf3* gene was amplified using a sense primer containing an *Eco*RI site and the ATG start codon (5′-GGGAATTCAGGAAACAGACCATGAGTTTTCCGGTTTGTCTCGTCA -  3′) and an antisense primer containing a *Hin*dIII site (5′-GGAAGCTTATGTGTTGAGCAGCAGCCCGxTTCTCGATCG  3′). The amplified DNA fragment was ligated into the *Eco*RI-*Hin*dIII site of pSE420D (Daicel Chemical Industries, Osaka, Japan) [18]. The resultant plasmid, pSORF3, was deposited in the International Patent Organism Depositary, National Institute of Advanced Industrial Science and Technology (Ibaraki, Japan) under accession number FERM-P-20287. To obtain recombinant ORF3, *E. coli* JM109 carrying pSORF3 was cultivated in LB medium containing 50 *μ*g/ml ampicillin and 0.1 mM IPTG at 37°C for 16 hours.

### 2.6. Purification of the orf3 Gene Product

The standard buffer used throughout purification was 10 mM potassium phosphate buffer (pH 8.0), and all operations were done at 4°C. Cultured *E. coli* cells expressing ORF3 were harvested by centrifugation, resuspended in 0.1 M potassium phosphate buffer (pH 8.0) containing 0.02% 2-mercaptoethanol (2-ME) and 2 mM phenylmethylsulfonyl fluoride (PMSF), and disrupted using a Micro Smash MS-100 (TOMY, Tokyo, Japan). After centrifugation, the supernatant was fractionated by ammonium sulfate precipitation (0–50% saturation). The enzyme-containing fraction was resuspended in 0.1 M potassium phosphate buffer (pH 8.0) containing 0.02% 2-ME and 2 mM PMSF, and dialyzed against the same buffer. The enzyme fraction was applied to a Q-Sepharose FF column (Pharmacia, Columbus, OH) equilibrated with the standard buffer containing 0.01% 2-ME. The enzyme was eluted with a linear gradient of 0–0.5 M NaCl in the same buffer. The enzyme fractions were collected, concentrated, dialyzed against the standard buffer containing 0.01% 2-ME and 20% saturated ammonium sulfate, and centrifuged. The supernatant was applied to a Phenyl superose HP 26/10 column (Pharmacia, Columbus, OH) equilibrated with the standard buffer containing 0.01% 2-ME and 30% saturated ammonium sulfate. The enzyme was eluted with a linear gradient of 20–0% saturated ammonium sulfate in the buffer. The enzyme fractions were collected, concentrated and dialyzed against the standard buffer containing 0.01% 2-ME. The final preparation of the enzyme was stored at −80°C until use.

### 2.7. Enzyme Assay

l-Phenylserine dehydrogenase activity was assayed by monitoring the increase in absorbance at 340 nm due to the production of NADH at 30°C in a 1-ml reaction mixture containing 20 mM dl-*threo*-*β*-phenylserine and 2.5  mM NAD^+^ in 0.2 M Glycine-KCl-KOH buffer (pH 10.5). d-Phenylserine dehydrogenase activity was determined as previously described [16].

### 2.8. Thin Layer Chromatography (TLC) Analysis

A reaction solution containing 40 mM dl-*threo*-*β*-phenylserine, 4.8  mM NAD^+^, and 0.3 mg/ml purified ORF3 in 0.1 M Glycine-KCl-KOH buffer (pH 10.5) was incubated overnight at 30°C. The reaction solution, dl-*threo*-*β*-phenylserine, and 2-aminoacetophenone were applied to a TLC plate, Kieselgel 60 F_254_ (Merck, Darmstadt, Germany). The chromatogram was developed using *n*-butanol-acetic acid-water (4:1:1, by vol.). The spots of dl-*threo*-phenylserine and 2-aminoacetophenone were detected by spraying the TLC plate with 1.5% ninhydrin solution in acetone-ethanol (7:3, by vol.) and incubating at 65°C until color developed.

### 2.9. Analytical Methods for Enzyme

Protein concentration was determined using a Protein assay kit (Bio-Rad, Hercules, CA) with bovine serum albumin as standard. The molecular mass of the subunit of l-phenylserine dehydrogenase was examined by SDS-PAGE using Protein Markers for SDS-PAGE (Nacalai Tesque, Kyoto, Japan). The molecular mass of native l-phenylserine dehydrogenase was estimated by HPLC on a TSK-GEL G3000SW column (*0* *.*75 × 60 cm) operating at room temperature. The column was eluted with 0.1 M potassium phosphate buffer (pH 7.0) containing 0.2 M NaCl at a flow rate of 0.7 ml/min. Amino acid sequences were obtained from PubMed at NCBI (http://www.ncbi.nlm.nih.gov/). A homology search was performed using the BLAST program [19] at GenomeNet (http://www.genome.ad.jp/). Multiple alignments were obtained with the ClustalW program [20] at GenomeNet (http://www.genome.ad.jp/).

### 2.10. Nucleotide Sequence Accession Number

The nucleotide sequence data have been deposited in the DDBJ/EMBL/GenBank nucleotide sequence databases under accession number AB499092.

## 3. Results

### 3.1. Identification of a Gene Encoding d-Phenylserine Dehydrogenase

**Figure 1:**
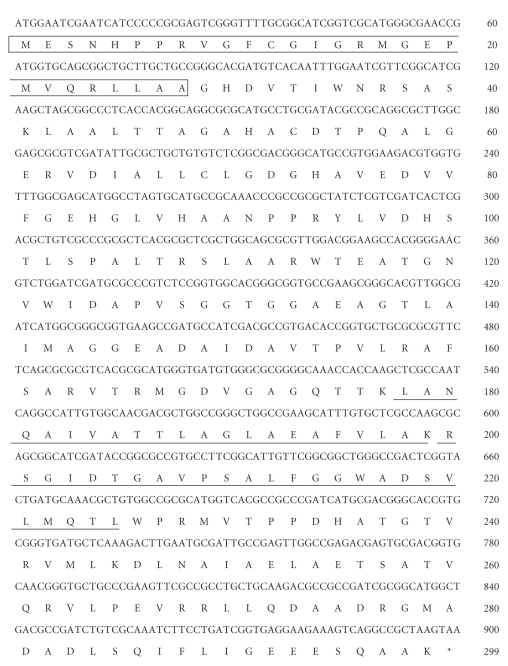
Nucleotide and deduced amino acid sequence of d-phenylserine dehydrogenase. N-terminal amino-acid sequence and internal amino acid sequences of the enzyme determined with an automated protein sequencer are boxed and underlined, respectively. The sequence data are deposited in the DDBJ/EMBL/GenBank database under the accession number AB499092.

Purified d-phenylserine dehydrogenase was obtained as previously described [16]. The enzyme was digested with lysyl endopeptidase, and the peptide products were purified by reversed-phase HPLC. The amino acid sequences of only two internal peptides could be determined (Figure 1). Based on the N-terminal amino-acid sequence and the internal amino acid sequences determined, an 897-bp nucleotide sequence was identified as the gene encoding d-phenylserine dehydrogenase (Figure 1). A crude extract of *E. coli* JM109 transformed with the pUPsDH expression vector containing the gene showed d-phenylserine dehydrogenase activity (33 U/mg), while that of wild-type *E. coli* JM109 was inactive.

### 3.2. Gene Organization of Regions Upstream and Downstream of the d-Phenylserine Dehydrogenase Gene

**Figure 2:**
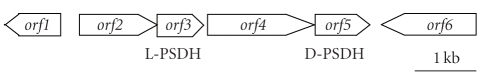
Genetic map around the d-phenylserine dehydrogenase gene.* orf1*: putative transcriptional regulator gene, *orf2*: putative MFS transporter gene, *orf3*: l-phenylserine dehydrogenase gene, *orf4*: putative dihydroxy acid dehydratase gene, *orf5*: d-phenylserine dehydrogenase gene, *orf6*: putative ABC peptide transporter gene.

To determine the nucleotide sequence of upstream and downstream regions of the gene encoding d-phenylserine dehydrogenase, inverse PCR was carried out. As a result, a 9,246-bp nucleotide sequence containing at least six open reading frames (ORFs) was determined (Figure 2). The transcriptional directions of *orf1* and *orf6* are opposite to those of the four other ORFs. Postulated promoter and terminator sequences are located immediately upstream of *orf2* and downstream of d-phenylserine dehydrogenase encoding* orf5, *respectively. These observations suggest that *orf2*, *orf3*, *orf4*, and *orf5* may form an operon.

*orf1* encodes a protein of 320 amino acids that is similar to amino acid sequences of putative LysR-type transcriptional regulators. Thus, *orf1* probably plays a role in the regulation of transcription of the operon. *orf2* encodes a protein of 436 amino acids that shows sequence similarity to putative major facilitator superfamily (MFS) transporters. *orf4* encodes a protein of 579 amino acids that is similar to amino acid sequences of putative dihydroxy acid dehydratases (ilvD). The *ilvD* gene has previously been identified in the *ilv* operon involved in branched-chain amino acids biosynthesis [21–23]; however, the operon containing the gene for d-phenylserine dehydrogenase did not contain other genes related to branched-chain amino acids metabolism. *orf5* encodes d-phenylserine dehydrogenase, which was previously characterized [16]. *orf6* encoded a protein of 520 amino acids that showed high similarity with amino acid sequences of putative ABC peptide transporters.

*orf3* encodes a protein of 259 amino acids that shares 37% identity with ketoreductase (RED2) from *Streptomyces violaceoruber *Tü22 [24] and 28% identity with 1,3,8-trihydroxynaphthalene reductase (3HNR) from *Magnaporthe grisea* (Figure 4) [25, 26]. The amino acid sequence of ORF3 also shows high similarity to that of putative short chain dehydrogenases and putative 3-oxoacyl-(acyl-carrier protein) reductases and 24% identity with serine dehydrogenase from *Agrobacterium tumefaciens* ICR 1600 [27]. A common GXXXGXG sequence, which is characteristic of an NAD(P)-binding site conserved in serine dehydrogenase and its homologs [27], was found in the N-terminal region of ORF3. For these reasons, we assumed that ORF3 has dehydrogenase activity, and considered that 3-hydroxy amino acids were likely to serve as a substrate for the enzyme, so cloning of *orf3* was done.

### 3.3. Purification of l-Phenylserine Dehydrogenase (ORF3)

ORF3 was purified to homogeneity from the recombinant *E. coli* JM109 cell carrying pSORF3. ORF3 has a calculated molecular mass of 27498.3 Da. The purified protein gave a single band with a molecular mass of 27 kDa on SDS-PAGE. The molecular mass of the native protein was determined to be 98 kDa by gel filtration. Because the elution of ORF3 was likely slightly slowed by nonspecific hydrophobic and ionic interactions between ORF3 and the gel filtration resin, the apparent molecular mass of the protein was most likely an underestimate. Therefore, ORF3 probably consists of four identical subunits. A summary of the specific activity and recovery of ORF3 during purification is shown in Table 1.

### 3.4. Properties of l-Phenylserine Dehydrogenase (ORF3)

**Scheme 2:**
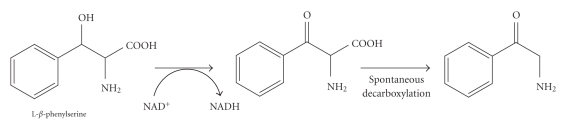
l-Phenylserine dehydrogenase reaction.

The molecular characteristics of the enzyme are shown in Tables 2, 3, and 4. The enzyme was significantly inhibited by 0.05 mM *p*-chloromercuribenzoate and 0.01 mM HgCl_2_. However, thiol reagents, such as *N*-ethylmaleimide and iodoacetamide, the chelating agent EDTA, and bivalent metal cations did not affect the enzyme (Table 2). The enzyme acted in an NAD^+^-dependent way on dl-*threo*-*β*-phenylserine but not on d-*threo*-*β*-phenylserine. Because we could not obtain pure l-*threo*-*β*-phenylserine, we were unable to perform enzyme assays with l-*threo*-*β*-phenylserine as a substrate. However, the data we obtained indicate that the enzyme showed activity towards only the l-form. The enzyme also acted on dl-*erythro*-*β*-phenylserine and dl-*threo*-(2-thienyl)serine. Pure l-forms of these compounds are also unavailable, but the enzyme likely acted on only the l-forms of *erythro*-*β*-phenylserine and *threo*-(2-thienyl)serine. Other amino acids tested did not serve as a substrate. The enzyme showed weak activity toward (*S*)-phenylethanol (Table 3). TLC analysis revealed that the enzyme converted l-*β*-phenylserine (*R_f _* = 0.52) into 2-aminoacetophenone (*R_f_* = 0.63). Therefore, we considered that the enzyme catalyzed the oxidation of the *β*-hydroxyl group of l-*β*-phenylserine and that the reaction product, l-*α*-amino-*β*-keto-*γ*-phenylpropionate, spontaneously decarboxylated to form 2-aminoacetophenone (Scheme 2). The enzyme preferred NAD^+^ to NADP^+^ as a coenzyme. The enzyme showed maximal activity at pH 11.2 and was stable between pH 6.1 and 11.2 at 30°C. The enzyme was stable at temperatures lower than 55°C for at least 10 minutes and showed the highest activity at 40°C (Table 4). The apparent *K*_*m*_ values for dl-*threo*-*β*-phenylserine and NAD^+^ were 59 and 2.1 mM, respectively.

## 4. Discussion

**Figure 3:**
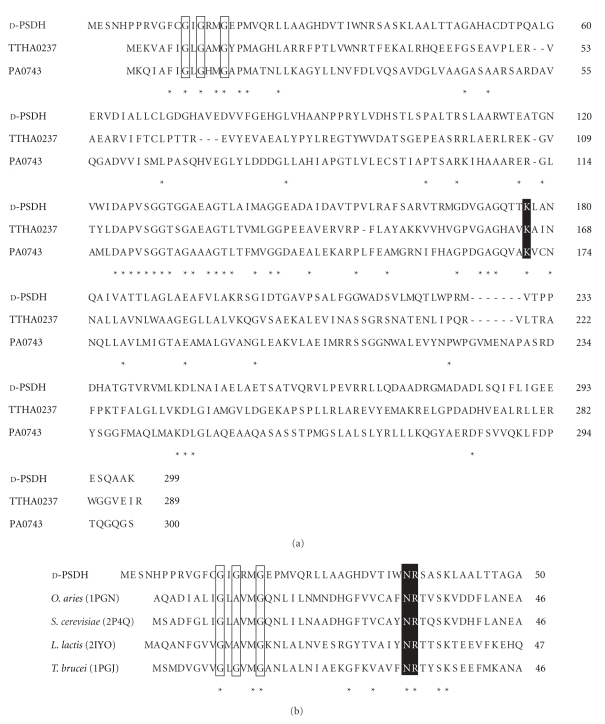
(a) Alignment of the amino acid sequence of d-phenylserine dehydrogenase (d-PSDH) with the sequences of 3-hydroxyisobutyrate dehydrogenase (TTHA0237) from *Thermus thermophilus* HB8 (PDB ID: 2CVZ) and 3-hydroxyisobutyrate dehydrogenase (PA0743) from *Pseudomonas aeruginosa* PAO1 (PDB ID: 3CUM). Conserved residues are marked with an asterisk and the key conserved catalytic residue is highlighted with a black background. The GXGXXG sequence fingerprint motif found in the Rossmann fold is boxed. (b) Alignment of the N-terminal amino-acid sequence of d-PSDH with the sequences of 6-phosphogluconate dehydrogenases from *Ovis aries* (PDB ID: 1PGN), *Saccharomyces cerevisiae* (PDB ID: 2P4Q), *Lactococcus lactis* (PDB ID: 2IYO), and *Trypanosoma brucei* (PDB ID: 1PGJ). Conserved residues are marked by an asterisk. The GX(G/A)XXG sequence fingerprint motif involved in coenzyme binding is boxed. The residues interacting with the 2′-phosphate group of NADP^+^ are highlighted with a black background. Accession numbers for the proteins used are as follows: d-PSDH, AB499092 (GenBank*™*); TTHA0237, Q5SLQ6 (TrEMBL); PA0743, Q9I5I6 (TrEMBL); and 6-phosphogluconate dehydrogenase from *O. aries*, P00349 (Swiss-Prot); *S. cerevisiae*, P38720 (Swiss-Prot); l *. lactis*, P96789 (Swiss-Prot); *T. brucei*, P31072 (Swiss-Prot).

The enzymological properties of d-phenylserine dehydrogenase have already been reported [16], but the nucleotide sequence of the gene encoding d-phenylserine dehydrogenase was determined in this work. The amino acid sequence of d-phenylserine dehydrogenase shares 24% identity with 3-hydroxyisobutyrate dehydrogenase (TTHA0237) from *Thermus thermophilus* HB8 [28] and 24% identity with a possible 3-hydroxyisobutyrate dehydrogenase (PA0743) from *Pseudomonas aeruginosa* PAO1. An alignment of the amino acid sequences of d-phenylserine dehydrogenase, TTHA0237, and PA0743 is shown in Figure 3. Many NAD/NADP-dependent dehydrogenases contain the Rossmann fold for nucleotide binding; the pyrophosphate group interacts with the GXGXX(G/A) motif found in the Rossmann fold [29]. This characteristic glycine-rich fingerprint motif was highly conserved in the N-termini of d-phenylserine dehydrogenase, TTHA0237, and PA0743. Similarly, alignment of the amino acid sequence of d-phenylserine dehydrogenase with the sequences of 6-phosphogluconate dehydrogenase from *Ovis aries* [30], *Saccharomyces cerevisiae* [31], *Lactococcus lactis* [32], and *Trypanosoma brucei* [33] showed that the GX(G/A)XXG motif and residues interacting with 2′-phosphate group of NADP^+^ (Gln36 and Arg37) were highly conserved among these enzymes. d-Phenylserine dehydrogenase and these 6-phosphogluconate dehydrogenases prefer NADP^+^ to NAD^+^ as a coenzyme. Moreover, a catalytic residue, Lys177, was also conserved in d-phenylserine dehydrogenase, TTHA0237, and PA0743.

**Figure 4:**
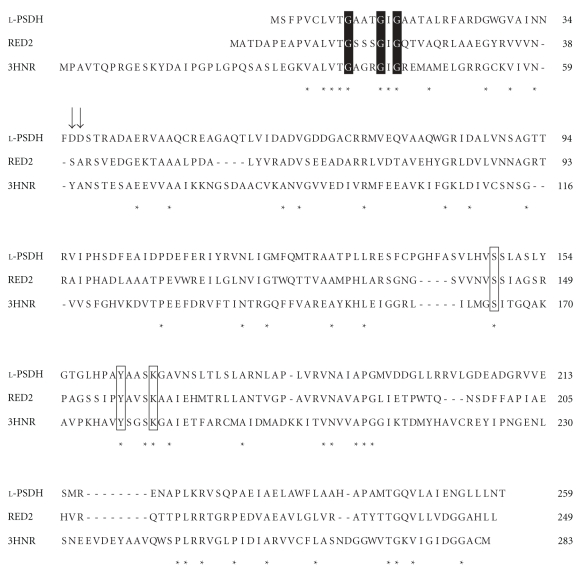
Alignment of the amino acid sequence of l-phenylserine dehydrogenase (l-PSDH) with the sequences of RED2 (ketoreductase) from *Streptomyces violaceoruber* Tü22 and 1,3,8-trihydroxynaphthalene reductase (3HNR) from *Magnaporthe grisea*. Conserved residues are marked by an asterisk. Residues forming the catalytic triad are boxed and the glycine-rich fingerprint motif is indicated with a black background. The residues Asp36 and Asp37 are shown by arrows. Accession numbers for the proteins used are as follows: l-PSDH, AB499092 (GenBank*™*); RED2, Q65YY6 (TrEMBL); 3HNR, Q12634 (Swiss-Prot).

The molecular characteristics of l-phenylserine dehydrogenase and d-phenylserine dehydrogenase are summarized in Table 4. The amino acid sequences of these enzymes showed no homology to each other and each enzyme belongs to a different protein family. The amino acid sequence of l-phenylserine dehydrogenase was similar to those of ketoreductase (RED2) from *Streptomyces violaceoruber* Tü22 [24] and 1,3,8-trihydroxynaphthalene reductase (3HNR) from *Magnaporthe grisea* [25, 26]. The amino acid sequences of l-phenylserine dehydrogenase and two homologs belonging to the short chain dehydrogenase/reductase (SDR) family aligned well (Figure 4). Members of the SDR family contain a similar structural fold, which shows a common nucleotide-binding site characterized by a GXXXGXG fingerprint motif [25]. Moreover, Arg or Asp residues located 18–20 residues downstream from the motif are responsible for nucleotide specificity [25, 34]. The characteristic glycine-rich fingerprint motif was conserved in the N-terminus of l-phenylserine dehydrogenase. Acidic residues, Asp36 or Asp37, which are 20 and 21 residues downstream, respectively, from the motif probably recognize the 2′-hydroxy group of NAD^+^. Our kinetic analysis also indicated that l-phenylserine dehydrogenase prefers NAD^+^ to NADP^+^ as the coenzyme. An X-ray structure of 3HNR complexed with NADPH and tricyclazole revealed that Ser164, Tyr178, and Lys182 compose the catalytic triad [25]. These residues were highly conserved in l-phenylserine dehydrogenase, RED2, and 3HNR (corresponding to Ser148, Tyr162, and Lys166 in l-phenylserine dehydrogenase) (Figure 4).

Although threonine [14, 35–37], serine [38, 39], and phenylalanine [40] serve as substrates for many enzymes acting on phenylserine, these amino acids were not accepted as substrates by l-phenylserine dehydrogenase. Among the amino acids tested, l-phenylserine and l-*threo*-(2-thienyl)serine were good substrates for l-phenylserine dehydrogenase. The genes encoding l-phenylserine dehydrogenase (ORF3) and d-phenylserine dehydrogenase (ORF5) were located within a single operon, and the reaction product of both enzymes is 2-aminoacetophenone. Moreover, d-phenylserine dehydrogenase is induced by addition of dl-*threo*-*β*-phenylserine to a culture medium as a sole source of carbon and nitrogen [16]. Therefore, we consider that d-phenylserine dehydrogenase acts physiologically on d-*threo*-*β*-phenylserine. For these reasons, we assume that the physiological function of l-phenylserine dehydrogenase is an NAD^+^-dependent conversion of l-phenylserine into 2-aminoacetophenone and carbon dioxide.

## Figures and Tables

**Table 1:** Purification of recombinant l-phenylserine dehydrogenase.

Step	Activity	Protein	Specific activity	Yield
	*units*	*mg*	*units/mg*	*%*
Crude extract	1400	1100	1.3	100
(NH_4_)_2_SO_4_ fractionation	1800	880	2.0	130
Q-Sepharose FF	1100	180	6.1	79
Phenyl-Sepharose	140	22	6.5	10

The enzyme activity was measured with 20 mM dl-*threo*-*β*-phenylserine and 2.5 mM NAD^+^ in 0.2 M glycine-KCl-KOH buffer (pH 10.5) at 30°C.

**Table 2:** Effect of various compounds on the activity of l-phenylserine dehydrogenase.

Compound	Concentration	Relative activity
	(mM)	(%)
None	—	100
*p*-Chloromercuribenzoate	0.05	0
*N*-Ethylmaleimide	1	89
Iodoacetamide	1	91
EDTA^a^	1	94
HgCl_2_	0.01	21
CuSO_4_	1	110
ZnSO_4_	1	92
MnSO_4_	1	110
MgSO_4_	1	100
BaCl_2_	1	99
NiCl_2_	1	95
CoCl_2_	1	94
CaCl_2_	1	93
KCl	187	87

l-Phenylserine dehydrogenase activity was determined in reaction mixtures containing the indicated additive, 20 mM dl-*threo*-*β*-phenylserine and 2.5 mM NAD^+^ in 0.2 M glycine-KCl-KOH buffer (pH 10.5) at 30°C.

^a^Ethylenediaminetetraacetic acid.

**Table 3:** Substrate specificity of l-phenylserine dehydrogenase.

Compound	Concentration	Relative activity
	(mM)	(%)
dl-*threo*-*β*-phenylserine	20	100
d-*threo*-*β*-phenylserine	10	0
dl-*erythro*-*β*-phenylserine	20	840
dl-*threo*-(2-thienyl)serine	20	530
(*S*)-phenylethanol	10	6.9
dl-hydroxynorvaline	20	0
l-threonine	10	0
d-threonine	10	0
l-*allo*-threonine	10	0
d-*allo*-threonine	10	0
l-serine	10	0
d-serine	10	0
l-phenylalanine	10	0
d-phenylalanine	10	0

l-Phenylserine dehydrogenase activity was determined in reaction mixtures containing 10 or 20 mM substrate, as indicated, and 1.0 mM NAD^+^ in 0.2 M glycine-KCl-KOH buffer (pH 10.5) at 30°C.

**Table 4:** Comparison of l-phenylserine dehydrogenase (l-PSDH) with d-phenylserine dehydrogenase (d-PSDH).

	l-PSDH	d-PSDH
Protein	short-chain	*β*-hydroxy acid
family	dehydrogenase	dehydrogenase
Molecular mass	98 kDa	70 kDa
Molecular mass of subunit	27 kDa	31 kDa
Number of subunits	4	2
Coenzyme specificity	NAD^+^	NADP^+^
Optimum pH	pH 11.2	pH 10.4
pH Stability	pH 6.1–11.2	pH 6.7–9.1
Thermostability	55°C or below	50°C or below
Substrate specificity	l-form	l-form
